# Effective implementation and adaptation of structured robotic colorectal programme in a busy tertiary unit

**DOI:** 10.1007/s11701-020-01169-1

**Published:** 2020-11-03

**Authors:** A. Thomas, K. Altaf, D. Sochorova, U. Gur, A. Parvaiz, Shakil Ahmed

**Affiliations:** 1 Department of Surgery, Royal Liverpool and Broadgreen University Hospitals NHS Foundation Trust, Prescot Road, Liverpool, L7 8XP UK; 2 Faculty of Health Science, University of Portsmouth, Portsmouth, UK

**Keywords:** Robotic surgery, Colorectal cancer, Colorectal surgery, Laparoscopic surgery

## Abstract

**Background:**

Safety and feasibility of robotic colorectal surgery has been reported as increasing over the last decade. However safe implementation and adaptation of such a programme with comparable morbidities and acceptable oncological outcomes remains a challenge in a busy tertiary unit. We present our experience of implementation and adaptation of a structured robotic colorectal programme in a high-volume center in the United Kingdom.

**Methods:**

Two colorectal surgeons underwent a structured robotic colorectal training programme consisting of time on simulation console, dry and wet laboratory courses, case observation, and initial mentoring. Data were collected on consecutive robotic colorectal cancer resections over a period of 12 months and compared with colorectal cancer resections data of the same surgeons’ record prior to the adaptation of the new technique. Patient demographics including age, gender, American Society of Anesthesiologist score (ASA), Clavien–Dindo grading, previous abdominal surgeries, and BMI were included. Short-term outcomes including conversion to open, length of stay, return to theatre, 30- and 90-days mortality, blood loss, and post-operative analgesia were recorded. Tumour site, TNM staging, diverting stoma, neo-adjuvant therapy, total mesorectal excision (TME) grading and positive resection margins (R1) were compared. *p* values less than or equal to 0.05 were considered statistically significant.

**Results:**

Ninety colorectal cancer resections were performed with curative intent from June 2018 to June 2020. Thirty robotic colorectal cancer resections (RCcR) were performed after adaption of programme and were compared with 60 non-robotic colorectal cancer resections (N-RCcR) prior to implementation of technique. There was no conversion in the RCcR group; however, in N-RCcR group, five had open resection from start and the rest had laparoscopic surgery. In laparoscopic group, there were six (10.9%) conversions to open (two adhesions, three multi-visceral involvements, one intra-operative bleed). Male-to-female ratio was 20:09 in RCcR group and 33:20 in N-RCcR groups. No significant differences in gender (*p* = 0.5), median age (*p* = 0.47), BMI (*p* = 0.64) and ASA scores (*p* = 0.72) were present in either groups. Patient characteristics between the two groups were comparable aside from an increased proportion of rectal and sigmoid cancers in RCcR group. Mean operating time, and returns to theaters were comparable in both groups. Complications were fewer in RCcR group as compared to N-RCcR (16.6% vs 25%). RCcR group patients have reduced length of stay (5 days vs 7 days) but this is not statistically significant. Estimated blood loss and conversion to open surgery was significantly lesser in the robotic group (*p* < 0.01). The oncological outcomes from surgery including TNM, resection margin status, lymph node yield and circumferential resection margin (for rectal cancers) were all comparable. There was no 30-day mortality in either group.

**Conclusion:**

Implementation and integration of robotic colorectal surgery is safe and effective in a busy tertiary center through a structured training programme with comparable short-term survival and oncological outcomes during learning curve.

## Introduction

Laparoscopic colorectal surgery has been shown to provide benefits over open surgery in terms of quicker recovery, reduced incidence of abdominal wall hernias, shorter length of stay, and improved post-operative pain relief with equitable oncologic outcomes [1–4]. However, laparoscopic surgical technique presents some limitations which include a prolonged learning curve, two-dimensional imaging, an unstable camera platform, limited instrument mobility, and poor ergonomics for operating surgeons. Robotic surgery overcomes some of the inherent issues of laparoscopy providing three-dimensional (3-D) imaging, a stable camera platform, enhanced dexterity, fluidity and increased range of movement which is more closely akin to wrist movements, particularly in the narrowly-spaced pelvis. Additionally a reduced fulcrum effect and better optics afforded by robotic systems, as opposed to laparoscopic, makes it a far superior and a more appealing platform to colorectal surgeons [5, 6]. Furthermore recent improvement in robotic operative techniques such as single-docking totally robotic approach, and advancement in technology such as integrated table motion (ITM), reduced size of camera to 8 mm diameter, redesigned new thinner patient cart, accessing multi-quadrant surgeries, performing irrigation, suctioning, stapling, and sealing devices improve the surgeons experience further and may ultimately translate to improved patient outcomes.

Despite advancement in technology and studies acknowledging robotic surgery safety and feasibility implementation and adoption in busy tertiary unit remains a challenge [7]. Several factors such as high cost to healthcare providers, capital investment, long learning curves, theatre time consumption, pressure on waiting lists, and potentially inferior clinical outcomes during learning curves as compared to well established laparoscopic and open surgical techniques, to name a few, make its adoption difficult for many institutions around the world. A learning curve is usually defined as achieving proficiency in a new set of skills or technique by surgeons to produce comparable outcomes [8]. In cancer resection surgery, it is usually measured in terms of operating time, morbidity, conversion rate, readmission rate, quality of specimen, and the lymph node retrieval [9]. Reported number of cases required to achieve proficiency in laparoscopic and robotic colorectal surgery varies between studies [10]. Learning curves in robotic colorectal surgery pose a different type of challenge as it requires not only a surgeon to learn new skills or techniques but it also relies on institutional adoption to new technology and hence there is an institutional learning curve [11].

Robotic training programmes have been well-established in other surgical specialties such as urology and cardiac surgeries with significant improvement in patient’s outcomes particularly in prostate cancer surgery [12]. However formal training in robotic colorectal surgery and its implementation and adoption in routine practice still remains challenging due to multi-quadrant operative field, variability in tumour size and location, prolonged learning curve and case-load required to achieve competency that could benefit patient’s outcomes. During the learning curve period of both the surgeon and the institution, there is a potential for poor patient outcomes and oncological compromises [12, 13]. A standard structured training programme, therefore, remains an imperative for safe integration and implementation to achieve comparable oncological and short-term outcomes particularly during the learning curve period.

We shared our experience of the safe implementation and adoption of robotic colorectal surgery applying a structured training model and standardized approach in a busy tertiary unit and compared short-term and oncological outcomes with previously established laparoscopic and open approaches.

## Methods

Liverpool University Hospital NHS Foundation Trust is a regional tertiary referral center for colorectal cancers and serves the population of 2.5 million covering a wide geographical area of Merseyside and Cheshire. The colorectal MDT receive on average 25 new colorectal cancers referrals every week and performed over 350 colorectal cancer resections per year. In addition it is tertiary regional referral center for anal cancer, small early rectal cancer and multi-visceral complex pelvic cancers.

### Robotic system and surgeon training

The da Vinci Si^®^ Surgical system (Intuitive Surgical Inc, Sunnyvale, CA, USA) was used for all robotic cases. Two colorectal consultants experienced in laparoscopic and open colorectal surgery with interest in robotic colorectal surgery were nominated for the training programme. Each surgeon required to do an online module and assessment for the robotic Si^®^ system followed by console built-in simulation training modules and achieved simulation competence scores for camera targeting, suturing, depth perception and non-tactile visual feedback. Each surgeon enrolled in a structured robotic colorectal programme run by the European Academy of Robotic Colorectal Surgery (EARCS). The training programme constituted case observations in a center of excellence, 2-days courses on animal and cadaveric models and in-house mentoring of first few cases. Global assessment score (GAS) form were used by trainer to provide feedback to trainees at the end of each procedure [14]. All supervised procedures were performed under the mentorship of a single trainer that makes the process relatively easier and consistent for learning. Video recordings for each supervised case were encouraged to have retrospective feedback to reduce the learning curve. In all case single-docking, flip arm technique of patient cart were used with port re-configuration for the pelvic part of procedure [14, 15].

### Data and patients’ demographics

Data were collected retrospective from a prospectively maintained electronic medical record database of robotic colorectal cancer resections (RCcR) performed from June 2019 to June 2020. Consecutive colorectal cancer resections from June 2018 to June 2019 performed by same two surgeons using non-robotic colorectal cancer resection (N-RCcR) techniques prior to the adaption of robotic techniques were recorded.

All patients were staged using appropriate imaging tool including computer tomography (CT), magnetic resonance imaging (MRI) and colonoscopy. Patients were discussed in the colorectal MDT and on average often twice before surgical intervention. Approval was obtained from hospital New Device and Technique committee for the use of Robot Da Vinci Si^®^ in colorectal cancer resections. Patients were informed about the introduction of new technique and were given information sheet and informed written consent was obtained prior to surgical intervention. Patient outcomes and complications were prospective collected in electronic registry.

All procedures followed were in accordance with the ethical standards of the responsible committee on human experimentation (institutional and national) and with the Helsinki Declaration of 1975, as revised in 2000.

Patients demographic including age, gender, body mass index (BMI), co-morbidities, previous abdominal surgery and American Society of Anesthesiologists (ASA) score were recorded. Tumour specific characteristics including tumour location, pre-operative TNM staging and neoadjuvant chemo- or chemoradiotherapy treatment was included. Operative data including type docking time, operation performed, technique of procedure (RCcR/N-RCcR), anastomoses, covering stoma, length of procedure, conversion to open, estimated blood loss were collated. Post-operative details were recorded including Clavien–Dindo grading complications, returns to theatre, unplanned critical care admissions, length of stay, 30-day mortality and readmissions. Post-operative oncological data were collated including TNM stage, lymph node yield, and circumferential resection margin (CRM), resection margins status (R0/R1), quality for mesorectal in specimens (mesorectal grading) and adjuvant therapy.

First five cases were done under the supervision of a proctor at the trainee hospital site. In these cases, trainees completed the surgery as first operating surgeon—with proctor intervening only when necessary. It is important to mention that the trainees go through intensive programme of time on-console, animal and cadaveric courses, online modules, case observation and visit to proctor’s hospital for two cases, before starting independent practice. Both trainee surgeons routinely perform colorectal cancer resections using laparoscopic surgery. They have performed > 200 laparoscopic cancer resections independently using minimal access surgery in their life time.

### Statistical analysis

IBM SPSS version 25 (SPSS Inc. Chicago, IL, USA) and Microsoft Excel version 16.38 (2019) were used for statistical analysis. Non-parametric data were reported as a median with range/IQR (interquartile range); means with SD (standard deviations) were used for parametric data. *p* values less than or equal to 0.05 were considered statistically significant.

## Results

**Fig. 1:**
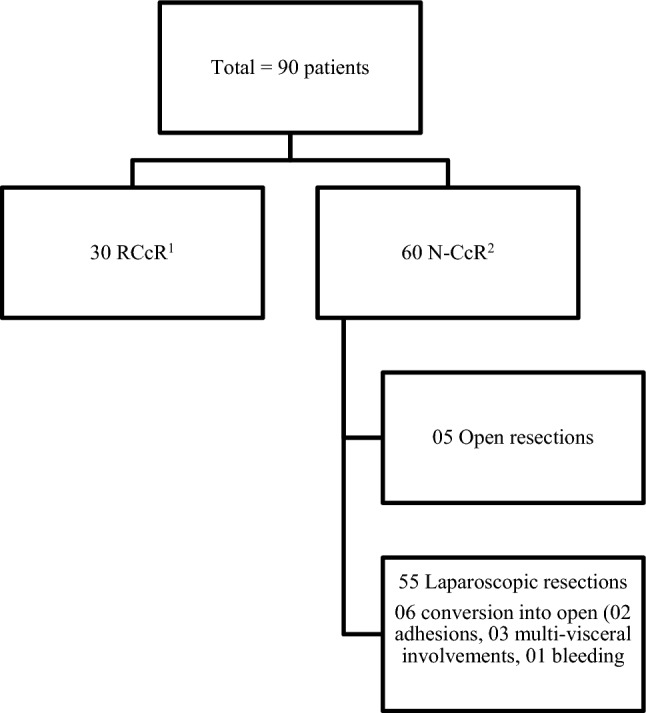
Flowchart of patients. ^1^*RCcR* robotic colorectal cancer resections. ^2^*N-RCcR* non-robotic colorectal cancer resections

Ninety colorectal cancers were performed with curative intent from June 2018 to June 2020. 60 cancers were resected using N-RCcR (laparoscopic or open) techniques from June 2018 to June 2019. In July 2019, RCcR technique was implemented and adopted in the unit and 30 cancers were performed within 12 months (Fig. 1). All 90 cases were performed by same two surgeons and were included in the study. Since the start of robotic programme, only six colorectal cancer resections have been performed non-robotically (five laparoscopic and one open) for various reasons (one panproctocolectomy, two extended right hemicolectomy, two previous left sided bowel resections and one high BMI > 55).

In N-RCcR group, five resections were performed using open techniques (three had multiple abdominal surgeries and two had large T4 tumours with involvement of small bowel and bladder) requiring multi visceral resection and 55 (91.6%) had laparoscopic procedures. In the laparoscopic group, there were six (10.9%) conversions to open (two for extensive adhesions, three for multi-visceral involvement; three small bowel and one bladder involvement; one for significant intra-operative bleeding).

In both RCcR and N-RCcR groups there was no statistical difference in age, gender (*p* = 0.5), median age (*p* = 0.47), BMI (*p* = 0.64) and ASA scores (*p* = 0.72) (Table 1). Nine patients in RCcR group and 21 in N-RCcR group had a history of previous abdominal surgery (*p* = 0.44) (Table 1).

**Table 1:** Patients demographics and characteristics

	RCcR, *n* = 30	N-RCcR, *n* = 60	*p*
Gender			
Male	20 (66.6%)	37 (61.6%)	0.5
Female	10 (33.3%)	23 (38.3%)	
Median age (years)	67	68	0.47
(Range)	(30–83)	(48–88)	
(IQR)	(61–71.5)	(59–74)	
Median BMI (kg/m^3^)	27.9	28.5	0.64
(Range)	(19.0–41.0)	(17.5–52.9)	
(IQR)	(24.6–30.5)	(24.1–32.0)	
ASA score			
I	1 (3.3%)	4 (6.6%)	0.72
II	17 (56.6%)	34 (56.6%)	
III	11 (38.0%)	19 (31.6%)	
IV	1 (3.3%)	3 (5.0%)	
Previous abdominal surgery	9 (31.0%)	23 (38.3%)	0.44
Neoadjuvant treatment	11 (37.9%)	8 (15.1%)	**0.01**
Pre-op T stage			
1	2 (6.6%)	1 (1.6%)	0.69
2	9 (30.0%)	21 (35.0%)	
3	15 (50.0%)	35 (58.3%)	
4	4 (13.3%)	3 (5.0%)	
Tumour site			
Rectum	23 (76.6%)	23 (38.3%)	**0.001**
Sigmoid	4 (13.3%)	13 (21.6%)	
Left colon	0	4 (6.6%)	
Transverse	0	2 (3.3%)	
Right colon	3 (10.0%)	18 (30.0%)	

Majority (90%) of tumours were in rectum and sigmoid colon in RCcR group as compared to N-RCcR group (60%). However, T staging between the groups were comparable (*p* = 0.69). In rectal cancers, there was no difference in the use of neo-adjuvant chemoradiotherapy between the two groups (11/23 in RCcR vs 10/23 in N-RCcR). All neo-adjuvant therapy included long-course radiotherapy in combination with chemotherapy followed by wait of 8–12 weeks before surgical intervention. In sigmoid cancers 2/4 patients in RCcR group had adjuvant chemotherapy and 4/13 had adjuvant chemotherapy in N-RCcR group. There was no neo-adjuvant therapy given in resection of right colon cancer resections.

In rectal cancer group, 05/23 had Abdominoperineal Resection of Rectum (APER) and 17/23 Anterior resections (AR) performed in RCcR group as compared to 04/23 APER and 14/23 AR in N-RCcR group. 04/30 sigmoid colon resections and 03/30 right colon cancer resections were performed using robot as compared to 13/60 sigmoid colon resections and 07/60 left colon resection and 20/60 right colon resections and 02/60 extended right colon resections in N-RCcR group (Table 2).

**Table 2:** Operative and post-operative outcomes for the groups

	RCcR, *n* = 30	N-RCcR, *n* = 60	*p*
Mean operative time (min)	239	233	0.62
(Range)	(135–430)	(134–369)	
(SD)	(59.83)	(59.54)	
Conversion to open	0/30	6/55 (10.9%)	**0.006**
Diverting stoma formation (rectum)	8/23 (34.7%)	6/23 (26.0%)	0.53
Estimated blood loss			
< 100 ml	29 (96.6%)	49 (81.6%)	**0.01**
100–500 ml	1 (3.3%)	6 (10%)	
> 500 ml	0	5 (8.3%)	
Complications (Clavien–Dindo classification)			
Grade I	2 (6.9%)	7 (13.2%)	0.27
Grade II	2 (6.9%)	4 (7.5%)	
Grade III	1 (IIIb) (3.4%)	2 (IIIb) (3.8%)	
Grade IV	0	0	
Grade V	0	1 (1.9%)	
Overall	5/30 (16.6%)	14/60 (23.3%)	
Return to theatre	1/30 (3.0%)	2/60 (3.3%)	0.94
Median length of stay (days)	5	7	0.26
(Range)	(3–54)	(3–77)	
(IQR)	(5–6.5)	(6–9)	
30-day readmissions	0/30	0/60	
30-day mortality	0/30	0/60	
Unplanned HDU/ITU admission	0/30	1/60 (1.6%)	

There was no significant difference in the mean operative time − 239 min, SD 59.83) in the RCcR group and 233 min (SD 59.54) in the N-RCcR group (*p* = 0.62). There was a significantly low incidence of conversion in the RCcR group as compared to N-RCcR group (*p* = 0.006). There was no statistical difference in formation of diverting stoma in both groups (08/23 in RCcR group vs 06/23 in N-CcR group) (*p* = 0.53). Estimated blood loss (EBL) was significantly lower in the RCcR group compared to the N-RCcR group (*p* = 0.01) (Table 2).

Overall morbidities were 5/30 (17.2%) in RCcR group and 14/60 (26.4%) in the N-RCcR group. In both the groups, the majority were grade I and II (as per Clavien–Dindo classification) and there was no statistically significant difference between the groups (*p* = 0.27). There were no 30-day readmissions or mortalities in either group. However, one patient died in the N-RCcR group after 77 days of laparoscopic converted into open left colon cancer resection. Patient had significant intra-operative bleed (1200 ml) which lead to an unplanned ITU admission. Later, patient was discharged to the ward but unfortunately, died of bilateral pneumonia.

One patient returned to theatre within 30 days due to an incarcerated parastomal hernia after APER in the R-CcR group. This was diagnosed before discharge and she then underwent small bowel resection and anastomosis. She made good recovery and was subsequently discharged. Two patients needed to return to theatre in the N-RCcR group (pelvic collection due to anastomosis leak which was drained through the rectum; wound dehiscence which required closure) (*p* = 0.94). Median length of stay was 2 days shorter in the RCcR group (5 days) as compared to N-RCcR group (7 days) but not a statistically significant difference (*p* = 0.26) (Table 2).

There were no statistical differences in the T and N staging between the two groups (*p* = 0.2) (Table 3). Six patients in the N-RCcR group had documented metastatic disease in the liver at the time of presentation and were treated with liver resection, ablation or chemotherapy as compared to two patients in RCcR group. No patient in the RCcR group was found to have positive resection margin, as compared to two patients in the N-RCcR group (*p* = 0.9).

**Table 3:** Oncological outcomes of the groups

	Robotic group *n* = 30				Open/lap group *n* = 60			*p* values
T stage	Rectal	Colon	Total		Rectal	Colon	Total	0.24
T0	1	0	1		0	0	0	
T1	5	0	5		1	1	2	
T2	5	3	8		9	4	15	
T3	11	4	15		12	24	36	
T4	1	0	1		1	8	9	
N stage								
N0	17	5	22		14	17	31	
N1	5	1	6		8	12	20	
N2	1	1	2		1	8	9	
M stage								
M0	23	7	30		22	32	54	
M1	0	0	0		1	5	6	
Resection margin status								0.9
R0	23	7	30		23	35	58	
R1	0	0	0		0	2	2	
CRM								0.08
Mesorectal fascia	22/23				18/23			
Intra-mesorectal	1/23				5/23			
Median number of lymph nodes	16				17			0.9
(Range)	(3–53)				(5–38)			
(IQR)	(14–22)				(13–23)			
Adjuvant therapy	4/30 (13.3%) 4 Chemotherapy				28/60 (46.6%) 27 Chemotherapy 1 Liver lesion ablation			**0.0001**

Grading of TME specimen in rectal cancer resections were all (22/23) reported as mesorectal fascia (previously mesorectum grade 3) except one patient in RCcR group, as compared to 18/23 in the N-RCcR group. The difference in the TME mesorectum specimen grading between the groups did not reach statistical significance (*p* = 0.08). There was no statistically significant difference between the RCcR (median 17 lymph node) and N-RCcR (median 16 lymph node) groups (*p* = 0.9).

**Fig. 2:**
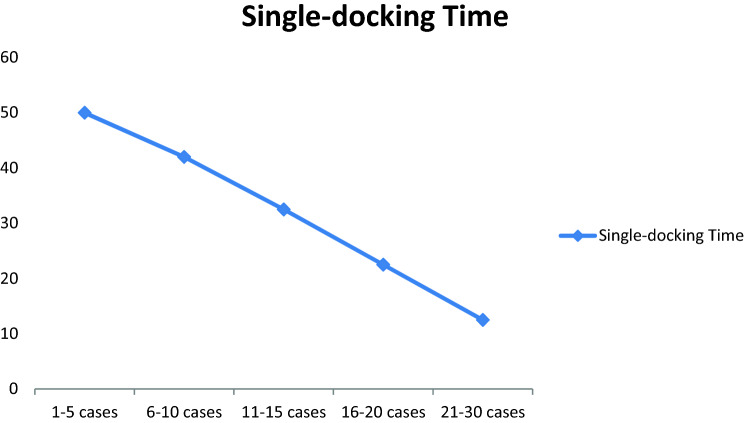
Single-docking time taken in first five cases, 6–10th cases, 11–15th cases, 16–20th cases and 21st–30th cases. *Y* axis showed time in minutes

Single-docking were used from the start and our experience demonstrated reduction in docking time as the learning curve progressed (Fig. 2).

## Discussion

Our study describes the experience of a single center university hospital in adopting a new surgical platform, associated considerations, and difficulties encountered. We did not see any difference between our groups of patients in terms of complications, return to theatre, 30-day readmissions, oncological outcome or mortality. This is consistent with published literatures [2, 16–19]. Although RCcR group was found to have shorter LOS, it did not reach statistical significance due to small number of cases. We also did not identify an increase in operative time duration in RCcR group which has been highlighted as a concern with robotic surgeries in some recent studies [20, 21]. This is usually attributable to the time required to dock the robot which may have to be repeated during the procedure when exploring different quadrants of the abdomen. We used single docking complete robotic approach that reduced mean operative times, therefore, did not impact on overall productivity of theatres and unit. There was no conversion to laparoscopic or open approaches in our robotic group, similar to previous published studies [20, 21]. The conversion rate in our N-RCcR cohort was also consistent with previous published literature [23, 24]. Our experience showed less EBL in RCcR group which is consistent with extant literature [25, 26]. In colorectal cancer surgery, reducing blood loss and henceforth, transfusion peri-operatively has been recognized to reduce morbidity and improve long-term survival [27–29]. We observed a higher number of defunctioning stoma in our RCrR group compared with the N-RcrR group. Similar to previous studies, this is likely attributable to the fact that more rectal resections took place in the robotic group [16, 22, 30]. Our study showed no significant differences in oncological outcomes between the two groups in terms of resection margin status, median lymph node yield, and CRM for rectal cancers, which is again consistent with the recently published meta-analysis [31].

It is important to recognise that the introduction of a new technically challenging platform into routine clinical practice requires a structured approach, an intense training programme, close supervision, and vigorous audit tools. But more importantly, it requires critical understanding of the concept and impact of the learning curve, which is determined by the number of cases needed to reach competence and denotes the rate of progress in acquisition of a given technical skill, which is expected to improve over time. This is followed by a plateau which is indicative of limited room for improvement [33]. Although the learning curve is usually surgeon dependent and robotic-assisted resections rely on many variables inherent to the institution, there is no agreed number of cases required to achieve competency in laparoscopic and robotic colorectal resections [10]. Literature has already informed us that in the context of robotic colorectal surgery, previous laparoscopic experience helps to ‘flatten’ the learning curve and leads to a shorter learning process when transitioning to the robotic platform. It also informs us of the number of cases or duration of time needed for an individual laparoscopic surgeon to achieve equivalent performance level in robotic surgery [34–37]. It is safe to deduce from existing literature that introduction of a robotic system into a specialist colorectal unit, first and foremost, requires the establishment of a robotic colorectal cancer surgery program with a high volume of cases. This integration may not significantly affect the duration of surgical procedure, various proficiency targets, and other short-term outcomes [38] and that many of the previously acquired skills are easily transferrable to a new technique [36]. We have inferred the same from our experience of introducing robot-assisted system to our clinical practice. It must be said, however, that the learning curve doesn’t only pertain to the surgeon but also includes the experience of the entire theatre team and the scrub nurses.

Another point to consider is the place and value of the training programme in helping with the learning curve. Studies have shown that a well-constructed and organised in-house robotic training programme can help a new department and team overcomes the learning curve faster [36, 37]. This should be augmented with attending other specific training programmes to enhance the previously acquired laparoscopic skills and expedite the process of learning. A study reported that institutional training programmes had a significant impact on the learning curve, with less number of cases needed to overcome the curve when surgeons had participated in such programmes [38]. But what this training programme should actually involve is up for debate. In recent times, technological advances like feedback loops are being encouraged where personalised recommendations are made to improve and shorten the learning curve [39].

Current evidence suggests that presence and active participation of an experienced proctor can advance the program by establishing a system that ensures a faster learning curve institutionally, including standardisation of patient and robot positioning and docking, port placement, appropriate use of instrumentation, and avoidance of arm collision and failure of instruments. It is critical to realise that this has to be treated as a team effort and a team learning experience. Therefore, it is equally important to integrate the operating theatre team into the system [40, 41]. Improved and faster learning curves are not just the result of a single surgeon’s proficiency but reliant on the overall efficiency of an entire team. Efforts should be concentrated and focused on training a team, rather than individuals if a new service is to succeed [42]. Experienced and specifically trained for purpose robotic surgical team is paramount for successful integration of robotic platforms into current surgical practice.

Our study doesn’t come without limitations. The increased proportion of rectal cancers within our robotic group does indicate a degree of selection bias. The anatomical confines of the pelvis, particularity for males remains a technical challenge in the treatment of rectal cancers and therefore more suited for robotic surgery [32]. One must be cognizant of the value of selection bias when evaluating a new technique as relatively ‘easy’ cases which have potentially favorable technical and clinical features are preferentially chosen initially. Therefore, we matched our laparoscopic cases to the consecutive robotic cases ensuring that the laparoscopic reference cases theoretically could have been operated by robotics at the same stage of training, thereby minimizing the selection bias. Other limitations of our study include the retrospective nature of case assessment, small number of patients, and limited number of surgeons performing robotic surgery in our center.

In short, efforts should be made on a wider level to help new centers establish these platforms through a national initiative like mentorship schemes, training fellowships, and other measures that would reduce the learning curve and gets the service running at its best in much shorter time. It may be appropriate for the widely influential platforms like The Association of Coloprotocology of Great Britain and Ireland Robotic Colorectal Surgery Registry and OCCTOPUS to consider to implement the same idea across UK. Only by having such a collaborative approach, the ecosystem could play a greater role in helping surgeons, trainees, institutions, and patients.

## Conclusions

Our experience demonstrates that instituting robotic colorectal surgery in a tertiary colorectal unit is safe and effective, providing equivalent short-term and oncological outcomes to standard operative techniques. Previous experience in laparoscopic surgery is transferrable to robotic surgery and helps to shorten the learning curve. Success of a well-run robotic colorectal programme depends on the adoption of structured training curriculum, efficacy of a dedicated team, institution’s support and many other variables that extend beyond the training console. These are the key elements in successfully integrating a technically challenging and complex programme like a robotic platform into a single center.

## Data Availability

Corresponding author is incharge of maintaining the regional prospective TEMS database. All of the data presented in this paper has been derived from it.
